# Advanced Combination Therapy for Pediatric Isolated Upper Gastrointestinal Crohn's Disease

**DOI:** 10.14309/crj.0000000000002407

**Published:** 2026-11-13

**Authors:** Noah Towbin, Thomas Huebner, Ronen Arai

**Affiliations:** 1 Emory University School of Medicine, Atlanta, GA; 2 Integrated Cellular and Molecular Diagnostics, Greenbelt, MD; 3 GastroHealth, Coral Springs, FL

**Keywords:** advanced combination therapy, biologic therapy, Crohn's disease, inflammatory bowel disease, pediatrics

## Abstract

Isolated upper gastrointestinal (UGI) Crohn's disease (CD) is uncommon and frequently presents nonspecifically, leading to diagnostic delay. We present, a 15-year-old adolescent boy with family history of ileal CD presented with nausea, vomiting, weight loss, and alternating diarrhea and constipation. Endoscopy with histology demonstrated isolated UGI CD. Advanced combination therapy with infliximab and upadacitinib was initiated. The patient's symptoms resolved and endoscopic mucosal improvement and histologic normalization occurred within four months. Following a taper of upadacitinib and continued infliximab, the patient remains in remission. This case supports use of advanced combination therapy for UGI CD.

## INTRODUCTION

Crohn's disease (CD) is a chronic inflammatory bowel disease that often manifests with discontinuous, transmural inflammation typically in the small bowel and/or colon, although it can present anywhere along the gastrointestinal tract. While ileocecal disease is most common, isolated upper gastrointestinal (UGI) inflammation occurs in 8%–10% of cases.^1^

Advanced therapy, defined as biologics or oral immunosuppressive agents, at diagnosis of Crohn's disease has become the standard of care to prevent disease progression and reduce complications. However, since about one-third of patients do not adequately respond to advanced monotherapy and half see diminished responses over time, advanced combination therapy (ACT) of a biologic with either a second biologic or an oral immunosuppressant has been studied and increasingly used in recent years.^2^

Traditional first-line ACT includes an anti-tumor necrosis factor agent such as infliximab (IFX) in combination with a thiopurine such as azathioprine (AZA). Such therapy is superior to monotherapy with either agent in achieving clinical improvement, remission, and mucosal healing.^3^ Given the time needed to titrate thiopurines to therapeutic doses while avoiding bone marrow toxicity, and their association with lymphoma in young males when used with IFX, a need exists for more rapidly effective and less toxic medication combinations.^4^

This is a case of new-onset isolated UGI CD treated aggressively with a novel ACT to achieve early healing and prevent complications.

## CASE REPORT

Patient was a 15-year-old adolescent boy presenting in March 2025 with 3 months of intermittent nausea and vomiting, 10-pound weight loss (body mass index 16.9), and diarrhea alternating with constipation. His father had ileal stenotic CD.

He was referred to a pediatric gastroenterologist. Esophagogastroduodenoscopy (EGD) in August 2025 demonstrated severe antral gastritis with erosions, friability, granularity, and the scope could not pass beyond the pylorus due to a stricture. Histology of the antrum demonstrated sparse giant cells. Colonoscopy with ileoscopy was normal, with unremarkable histology in the colon and ileum. Abdominal ultrasound and MR enterography were normal. Capsule endoscopy was not performed because the patient had a pyloric stricture.

The patient was referred to an adult inflammatory bowel disease expert. Based on review of the prior EGD, empiric prednisone (20 mg daily) was initiated. This dose had a minimal effect on his symptoms. Repeat EGD in October 2025 showed disease progression with distal esophageal aphthous ulcers, discontinuous severe antral inflammation, longitudinal incisural ulcers, and a pyloric stricture that could not be passed (Figure 1). Biopsy of all sites showed non-necrotizing granulomata and increased intraepithelial lymphocytes, consistent with CD (Figure 2). Laboratory test results included Hgb 10.4 g/dL (reference range, 12.0-16.9), mean corpuscular volume 82.9 fL (reference range, 79.4–99.7), platelets 681k (reference range, 140–400), ferritin 17 ng/mL (reference range, 11–172), albumin 3.9 g/dL (reference range, 3.6–5.1), C-reactive protein <3 mg/L (reference range, <8.0), and negative QuantiFERON-TB Gold Plus and hepatitis B surface antigen.

**Figure 1.**
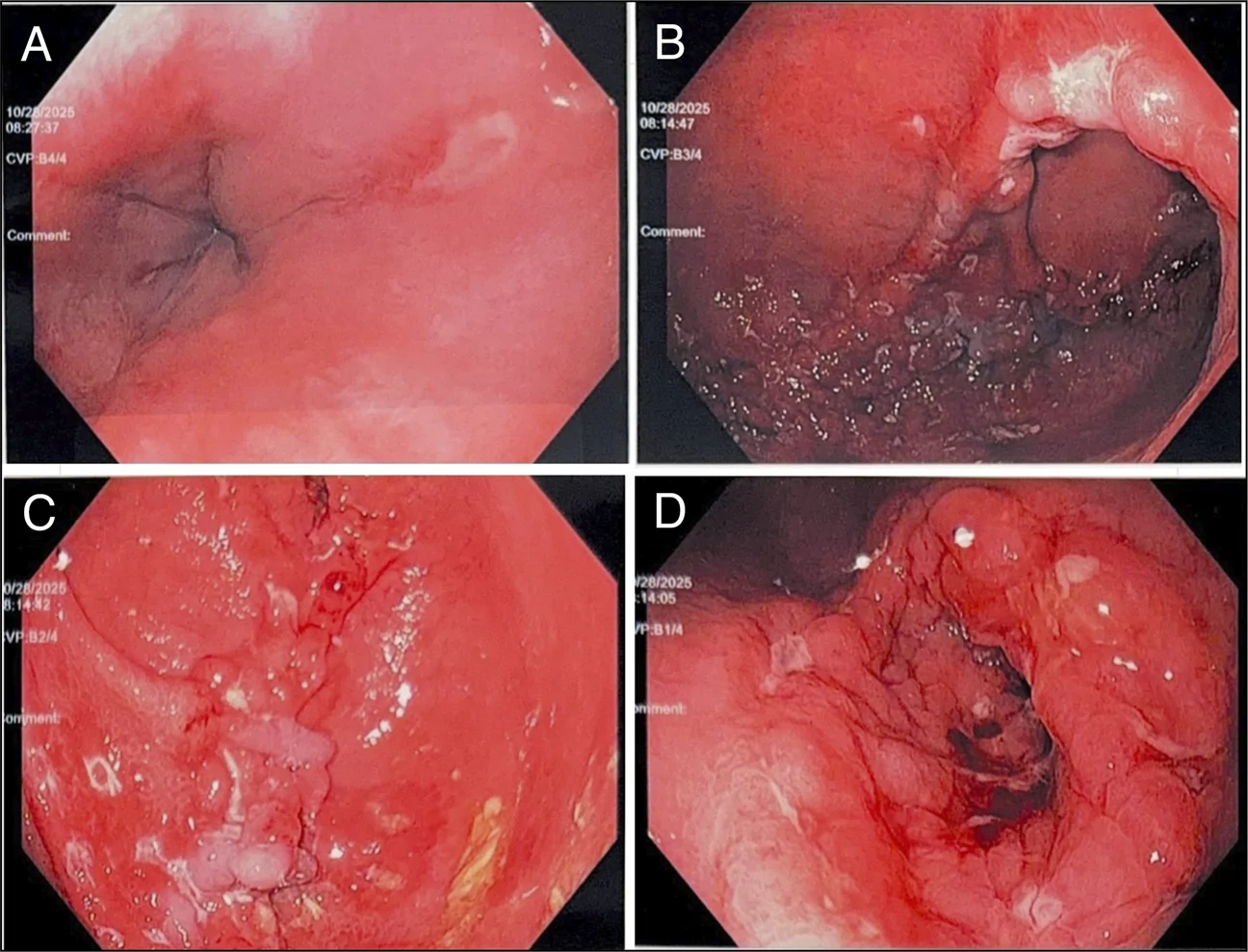
Esophagogastroduodenoscopy images from October 2025 (A) esophagus; (B) gastric body; (C) gastric antrum; (D) pylorus.

Combination IFX and AZA was considered but ultimately rejected after discussing the lymphoma risk with the patient's parents.^4^ Instead, shared decision-making led us to select dual therapy with IFX and upadacitinib (UPA) (JAK1 inhibitor) in an effort to achieve rapid disease control and healing while avoiding the prolonged time to maximum effect associated with thiopurine use. Dietary therapy was not used. Due to difficulty swallowing pills, the patient was prescribed liquid UPA at an induction dose of 12 mg twice daily for 12 weeks given his age and weight. Prednisone was increased to 40 mg daily at this time while awaiting insurance approval.

While awaiting medication insurance approval and still on prednisone, the patient was briefly hospitalized in November 2025 for persistent vomiting and abdominal pain. Abdominal computed tomography showed gastric wall thickening with mucosal hyperenhancement and submucosal edema. Intravenous steroids were initiated and he received the first induction IFX infusion (5 mg/kg) in the hospital. He continued standard induction and maintenance infusions after discharge along with prednisone. UPA was approved by insurance in mid-December 2025 and prednisone was tapered off.

At office follow-up in February 2026, the patient had resolution of all gastrointestinal symptoms. EGD a week later showed normal esophageal mucosa, resolution of all ulcers, gastric scarring with small pseudopolyps, and a persistent pyloric stricture that was easily balloon-dilated to 10 mm, allowing passage of the scope into the duodenum (Figure 3). The duodenal bulb appeared scarred and foreshortened, while the second portion appeared normal. Biopsies revealed reactive gastropathy without granulomas and regenerative changes in the duodenum.

**Figure 2.**
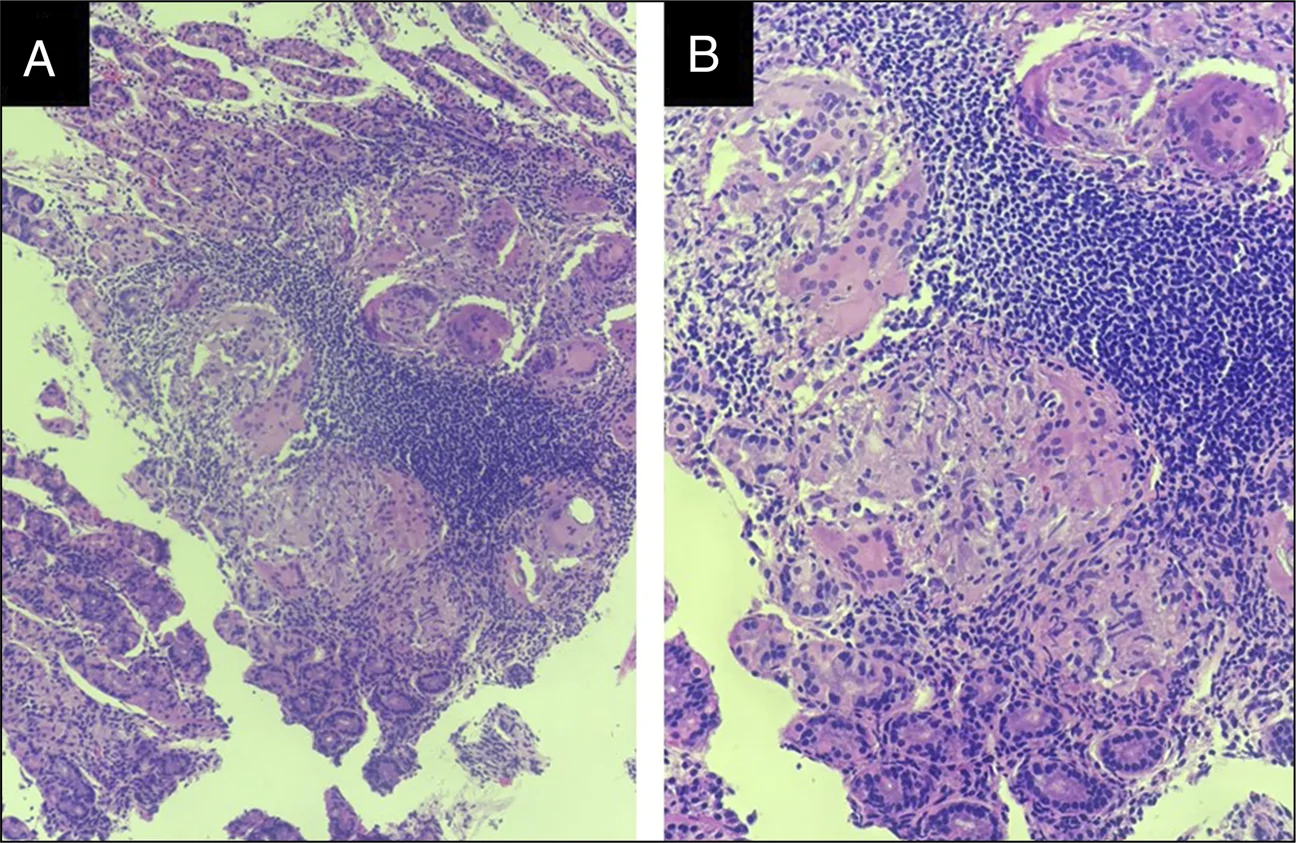
H&E stain from gastric biopsy taken in October 2025 (A) 100× magnification; (B) 200× magnification.

Based on these results, UPA was tapered by 5 mg every 2 weeks and discontinued by April 2026. At follow-up in May 2026, the patient felt well, was eating an unrestricted diet, had gained weight (BMI 19.3, from low of 14.7), and had regained his energy and quality of life. As of July 2026, laboratory test results included Hgb 11 g/dL, mean corpuscular volume 82.5 fL, platelets 314k, ferritin 7 ng/mL, albumin 4.2 g/dL, and C-reactive protein <3 mg/L. He continues maintenance IFX infusions.

## DISCUSSION

This is a case of pediatric CD presenting with isolated UGI involvement including a pyloric stricture at diagnosis.

The incidence of CD in the pediatric population is increasing, with around 20%–25% of patients presenting younger than age 18.^5^ Pediatric CD is often severe at presentation, demonstrating a more aggressive disease course with growth retardation, delayed puberty, increased fibrostenotic and penetrating complications, and psychological morbidity.^6^

The Montreal classification system includes disease of the esophagus, stomach, duodenum, jejunum, and proximal ileum in the L4 phenotype, although this is typically found along with ileocolonic disease.^1^ Isolated L4 CD has a low prevalence of 8%–10% of adult cases with limited data in pediatric populations.^1^ UGI CD often presents nonspecifically, leading to delayed diagnosis, advanced disease, and complications at presentation.

In an effort to achieve rapid healing and prevent further complications, ACT with IFX and UPA was used in this case.^3^ The patient was not started on the more traditional standard combination therapy for IFX/thiopurine due to the slower onset of action of AZA and its association with lymphoma in young males when combined with IFX.^7^ Prospective studies in newly diagnosed CD patients and retrospective studies on the effectiveness and safety of early ACT have demonstrated statistically significant increases in clinical remission, improved inflammatory markers, fewer disease flares, and reduced complications requiring surgery.^8,9^ Combinations being evaluated in ongoing trials include guselkumab (IL-23 inhibitor) with golimumab (anti-TNF) in the recently-presented DUET-CD trial, vedolizumab (anti-integrin) with UPA in the VICTRIVA trial, and vedolizumab with either ustekinumab (IL-12/23 antagonist) or adalimumab (anti-TNF) in the EXPLORER-2 trial.^9–13^

In this case study, a novel ACT using IFX and UPA resulted in rapid clinical remission, endoscopic improvement, and histologic disease resolution, supporting early effective therapy for management of UGI CD. Future randomized controlled trials are necessary to establish how well this combination compares with traditional ACT and newer emerging combination therapies (Figure 3).

**Figure 3.**
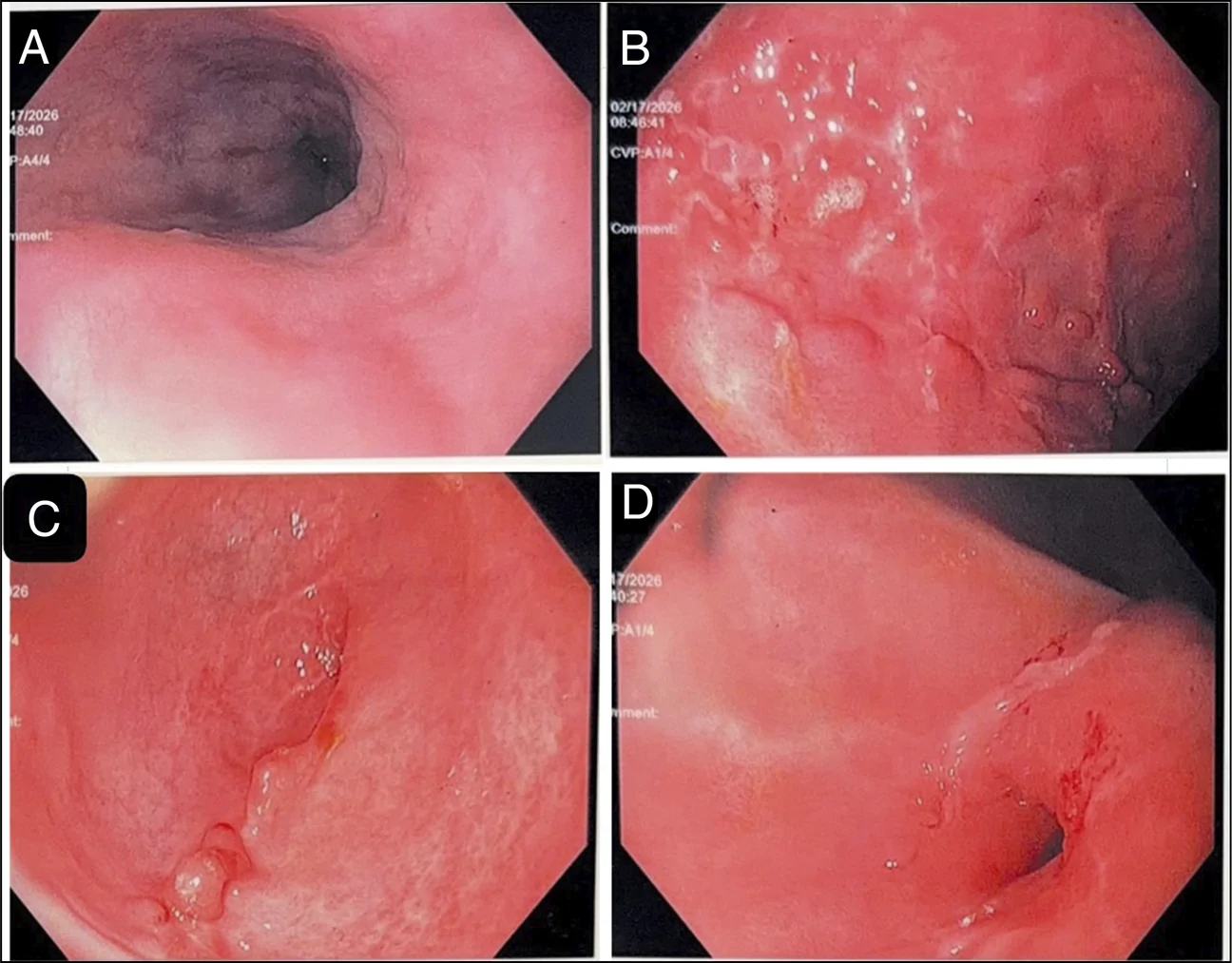
Esophagogastroduodenoscopy images from February 2026 (A) esophagus; (B) gastric body; (C) gastric antrum; (D) pylorus.

## DISCLOSURES

Author contributions: N. Towbin: review of patient documentation, manuscript drafting/editing. T. Huebner: review and contribution of patient histology slides. R. Arai: provision of care to the patient, and manuscript drafting/editing. R. Arai is the article guarantor.

Financial disclosure: None to report.

Previous presentation: Presented at the American College of Gastroenterology Annual Scientific Meeting, October 9–14, 2026; Nashville, TN.

Informed consent was obtained for this case report.

## ABBREVIATIONS:

ACT: advanced combination therapy
AZA: azathioprine
CD: Crohn's disease
EGD: esophagogastroduodenoscopy
IFX: infliximab
UGI: upper gastrointestinal
UPA: upadacitinib
